# Soil microbial carbon use efficiency differs between mycorrhizal trees: insights from substrate stoichiometry and microbial networks

**DOI:** 10.1093/ismeco/ycae173

**Published:** 2024-12-27

**Authors:** Jing Yu, Jingyi Yang, Lingrui Qu, Xiaoyi Huang, Yue Liu, Ping Jiang, Chao Wang

**Affiliations:** Chinese Academy of Sciences, Key Laboratory of Forest Ecology and Silviculture, Institute of Applied Ecology, Chinese Academy of Sciences, Shenyang 110016, China; University of Chinese Academy of Sciences, Beijing 100049, China; Chinese Academy of Sciences, Key Laboratory of Forest Ecology and Silviculture, Institute of Applied Ecology, Chinese Academy of Sciences, Shenyang 110016, China; Chinese Academy of Sciences, Key Laboratory of Forest Ecology and Silviculture, Institute of Applied Ecology, Chinese Academy of Sciences, Shenyang 110016, China; Chinese Academy of Sciences, Key Laboratory of Forest Ecology and Silviculture, Institute of Applied Ecology, Chinese Academy of Sciences, Shenyang 110016, China; University of Chinese Academy of Sciences, Beijing 100049, China; Chinese Academy of Sciences, Key Laboratory of Forest Ecology and Silviculture, Institute of Applied Ecology, Chinese Academy of Sciences, Shenyang 110016, China; Chinese Academy of Sciences, Key Laboratory of Forest Ecology and Silviculture, Institute of Applied Ecology, Chinese Academy of Sciences, Shenyang 110016, China; Chinese Academy of Sciences, Key Laboratory of Forest Ecology and Silviculture, Institute of Applied Ecology, Chinese Academy of Sciences, Shenyang 110016, China; Key Laboratory of Terrestrial Ecosystem Carbon Neutrality, Shenyang, Liaoning Province, 110016, China

**Keywords:** mycorrhizal type, microbial growth rate, stoichiometry, soil organic carbon, forest

## Abstract

The role of mycorrhizal associations in controlling forest soil carbon storage remains under debate. This uncertainty is potentially due to an incomplete understanding of their influence on the free-living soil microbiome and its functions. In this study, rhizosphere and non-rhizosphere soils were collected from eight arbuscular mycorrhizal (AM) and seven ectomycorrhizal (ECM) tree species in a temperate forest. We employed high-throughput sequencing and ^18^O-H_2_O labeling to analyze the soil microbial community and carbon use efficiency (CUE), respectively. We find microbial respiration rates are higher in rhizosphere than that in non-rhizosphere soils for ECM trees, whereas microbial growth rates show no significant differences. Consequently, microbial CUE is lower in rhizosphere compared to non-rhizosphere soils for ECM trees. In addition, we find that non-rhizosphere soils from ECM trees exhibited higher CUE compared to those from AM trees. Furthermore, we observe that bacterial–fungal co-occurrence networks in ECM soils exhibit greater complexity relative to AM ones. Using random forest and structural equation modeling analyses, we find that microbial stoichiometric carbon/nitrogen imbalance and network complexity are key predictors of soil microbial CUE for AM and ECM trees, respectively. Our findings shed new light on the pivotal role of mycorrhizal associations in shaping free-living microbial communities and their metabolic characteristics in the studied soils. These insights are critical for predicting soil carbon sequestration in response to shifts in ECM and AM species within temperate forest under climate change.

## Introduction

Soils constitute one of the largest carbon (C) reservoirs within terrestrial ecosystem and play a critical role in moderating climate change through CO_2_ uptake and release [1, 2]. Plant primary production, which is typically nutrient-limited, serves as the main sources of soil C [3, 4]. To overcome these limitations, >90% of terrestrial plants establish symbiotic relationships with mycorrhizal fungi, which facilitate the acquisition of nitrogen and phosphorus from the soil, alleviating nutrient constraints for their hosts [5, 6]. As a result, the presence and types of mycorrhiza are determinants of plant–soil C cycles and soil C storage [5].

Several studies have shown that ecosystems dominated by ectomycorrhizal (ECM) plants typically store more organic C in topsoil compared to those dominated by arbuscular mycorrhizal (AM) plants [7, 8]. However, this pattern may be reversed in deeper soil layers, where AM-dominated systems can potentially store more C [9]. Additionally, AM and ECM plants exhibit contrasting responses to global changes, which could significantly impact soil C storage [10]. For example, nitrogen deposition and warming tend to reduce soil C stocks in AM-dominated ecosystems, whereas ECM-dominated systems may experience increased C storage under similar conditions [11]. Despite these findings, the mechanisms by which mycorrhizal types influence soil C cycles remain debated.

Distinct differences in the free-living soil microbiome composition and function between AM and ECM systems likely play a critical role in soil C turnover [12]. Free-living soil microorganisms regulate soil C through two contrasting pathways: they release C via respiration during catabolic activities, while also contributing to C stabilization through the production of microbial residues [13, 14]. Emerging evidence indicates that microbial residues are a dominant source of stable soil organic C, and models incorporating microbial traits, including microbial necromass and carbon use efficiency (CUE), perform better in predicting soil C dynamics [15–17]. Microbial CUE—the efficiency with which microorganisms convert organic C into biomass rather than respiring it as CO_2_—is particularly important among biotic factors determining microbial necromass and soil C storage [18, 19]. Therefore, differences in microbial CUE may underlie the variations in soil C cycling and storage between AM and ECM systems. However, research directly addressing this topic remains limited [12].

The distinctions between AM and ECM system in terms of their associated plant litter quality, soil nutrient stoichiometry, and microbial community structure can potentially lead to variations in the microbial community CUE [5, 12]. AM plants tend to produce high-quality, easily decomposable litter compared to ECM plants [20, 21], which generate more recalcitrant litter with higher chemical resistance [22, 23]. These differences result in contrasting resource availability for microbial communities, with AM systems generally fostering higher microbial CUE due to efficient utilization of labile substrates [24]. In contrast, ECM systems often exhibit imbalanced C to nutrients stoichiometry and higher fungal-to-bacterial (F:B) ratios, which can constrain microbial growth efficiency and reduce CUE [25, 26]. Besides, a critical aspect of these differences lies in the distinct properties of rhizosphere and non-rhizosphere soils within AM and ECM systems. The rhizosphere, enriched with root exudates and labile organic substrates, supports active microbial communities but often promotes rapid turnover of C through microbial respiration [27]. In AM systems, the labile substrates in the rhizosphere may enhance microbial growth efficiency and CUE, while in ECM systems, the competition between ECM fungi and free-living microbes for organic N can limit microbial nutrient acquisition, leading to lower CUE [27]. In contrast, non-rhizosphere soils, with reduced labile C inputs, may exhibit slower microbial turnover and higher CUE due to the stabilization of microbial residues and lower metabolic costs [5]. This spatial variation highlights the interplay between root-microbe interactions and substrate quality in shaping microbial CUE.

In addition to these resource-driven differences, microbial network dynamics also play a critical role in influencing CUE. AM-dominated soils tend to harbor microbial communities with more diverse bacterial networks, facilitating synergistic interactions that promote resource sharing and enhance microbial efficiency [12, 28]. Conversely, ECM-dominated soils often feature fungal-dominated networks, in which competitive interactions for limited organic N among ECM fungi and decomposer microbes may increase metabolic costs and reduce CUE [25]. This difference in microbial network structure likely amplifies the disparities in CUE between rhizosphere and non-rhizosphere soils across AM and ECM systems. Taken together, while the understanding from previous studies indicates the differential in CUE between mycorrhizal types, the main drivers for these differences remain unknown.

To examine how the dominant mycorrhizal associations of different tree species influence soil microbial growth rate and CUE, we sampled rhizosphere and non-rhizosphere soil from 15 species in a natural Korean pine and broadleaf mixed forest, including eight AM plants and seven ECM plants. We used the ^18^O-H_2_O isotope labelling method to measure microbial CUE [18, 29, 30]. To explore the drivers of microbial CUE, we examined its associations with microbial diversity, network structure, and soil nutrient stoichiometry. We hypothesized that (i) microbial CUE will be lower in ECM-associated soils compared to AM-associated soils. This is due to ECM systems’ recalcitrant litter quality, nutrient imbalances, and fungal-dominated microbial networks, which are associated with higher metabolic costs and lower microbial CUE; (ii) microbial CUE will be higher in non-rhizosphere soils compared to rhizosphere soils in both AM and ECM systems. This is because non-rhizosphere soils, with less input of root exudates that often promote microbial activity, may experience reduced competition, thereby lowering metabolic costs and enhancing microbial CUE.

## Materials and methods

### Study area and soil sampling

The study was conducted in the Changbai Mountain Natural Reserve, Jilin Province, northeastern China (42.70°N, 127.63°E). This region has a temperate continental climate, with a mean annual temperature of 3.6°C and an annual precipitation of 700 mm. The elevation in this region ranges from 500 to 2700 m. The dominant plant species include *Pinus koraiensis*, *Abies holophylla Maxim*, *Acer barbinerve*, and *Tilia amurensis Rupr*. The study site was established in a Korean pine and broadleaf mixed forest at an elevation of 750 m, and field sampling was conducted in September 2019. Representative tree species from the study site were selected, including AM-associated species such as *Acer barbinerve*, *Acer mono*, *Acer pseudosieboldianum*, *Acer tegmentosum*, *Cerasus maximowiczii*, *Maackia amurensis*, *Malus baccata*, and *Syringa reticulata*, as well as ECM-associated species such as *Abies nephrolepis*, *Betula costata*, *Betula platyphylla*, *Larix gmelinii*, *Picea jezoensis*, *Pinus koraiensis*, and *Quercus mongolica*. Together, these selected species accounted for >60% of the total species in the study site.

Five individuals of each selected tree species were randomly chosen for soil sampling, collecting both non-rhizosphere and corresponding rhizosphere soil samples (*n* = 5). Soil collection followed the “adhering after shaking” method [31], where fine roots at the ends of coarse roots were gently shaken to remove loose soil. The detached soil was collected as “non-rhizosphere soil”, while the soil adhering to the fine roots was defined as “rhizosphere soil”. Roots with adhering soil were placed in plastic bags and stored in a dry ice-cooled container during field sampling. The samples were then transferred to the laboratory within 6 h. In the lab, soil was carefully brushed off the roots, and any remaining root debris was removed using tweezers. To preserve microbial community integrity, freshly sieved (<2 mm) non-rhizosphere and rhizosphere soil samples were stored at −20°C until analysis. Air-dried soil samples were used for determining soil chemical properties, while freshly incubated samples (~100 g) were pre-incubated at 15°C (the mean growing season temperature of the sampling site) for 24 h at 60% water-holding capacity (WHC) to assess microbial community metabolic properties.

### Soil physicochemical and microbial properties analysis

Soil water content was measured gravimetrically after oven drying for 24 h at 105°C. Soil WHC was determined by repeatedly saturating the soil with deionized water for 2 h, draining in a funnel with an ash-free cellulose filter paper for 6 h and then drying at 105°C for 24 h. Soil pH was measured in a 1:2.5 soil:water suspension using a pH electrode (PHS-3E, Leici, China). Soil microbial biomass carbon (MBC) and microbial biomass nitrogen (MBN) were determined by chloroform fumigation extraction method [32, 33]. Briefly, 20 g fresh samples were weighed in a 100 ml beaker, placed in a desiccator, and fumigated in the dark with alcohol-free chloroform for 48 h. We extracted fumigated and unfumigated soil samples using 0.5 M K_2_SO_4_ solution in a 1:4 soil-to-solution ratio and then measured the soil dissolved organic C (DOC) and total dissolved N (TDN) content of the filtrate using TOC analyzer (TOC-LCPH, Shimadzu, Japan). The content of MBC and MBN was calculated by dividing the difference between the DOC content and the TDN content in the extract of the fumigated sample and the corresponding non-fumigated sample by the conversion coefficient of 0.45 and 0.54, respectively [33].

We applied the ^18^O-H_2_O tracer incubation approach to estimate microbial CUE [30]. Briefly, 1 g of soil sample was weighed into a 2 ml brown sample bottle in duplicate, with one replicate adding ^18^O-H_2_O (98.0 at% ^18^O) to adjust the ^18^O abundance of soil water to 20 at% ^18^O and the other adding an equal volume of non-labelled water as a control treatment. After fully mixing, the sample bottles were transferred into 20 ml headspace vials and capped, then flushed with CO_2_-free air for 5 min to renew the gas in the headspace vials to limit the concentrations of CO_2_ to ~0 ppm. Three empty vials were set as negative controls for CO_2_ concentration analysis following the same procedure. The samples were incubated for 24 h at 15°C. After incubation, 10 ml gas sample was collected from each headspace vial with a syringe and the CO_2_ concentration was determined immediately with a gas chromatograph system (GC-7890B, Agilent, USA). Then the brown bottles containing soil were immediately retrieved and capped, frozen in a lyophilizer, followed by storage at −80°C until deoxyribonucleic acid (DNA) extraction.

Soil DNA was extracted using a DNA extraction kit (MoBio, PowerSoil) following the manufacturer’s procedures. The DNA concentration was determined by the Picogreen fluorescence assay (PicoGreen, Thermo Fisher) using a microplate spectrophotometer (Infinite M200, Tecan, Austria). The remaining DNA extract was dried in a silver capsule to subsequently determine the ^18^O abundance and total O content using IRMS-TC/EA (Thermo Fisher Scientific, USA). The newly DNA produced of soil samples during the 24-h incubation period (DNA_produced_, μg) was calculated as follows:


(1) \begin{equation*} {\mathrm{DNA}}_{\mathrm{produced}}={\mathrm{O}}_{\mathrm{total}}\ast \frac{\mathrm{at}{\%}_{\mathrm{excess}}}{100}\ast \frac{100}{\mathrm{at}{\%}_{\mathrm{final}}}\ast \frac{100}{31.21} \end{equation*}

where O_total_ is the total O content (μg) of the dried DNA extract, at%_excess_ is the difference between at% ^18^O of the labeled sample and at% ^18^O of the non-labeled sample, and 31.21 is the average percentage of O in DNA (C_39_H_44_O_24_N_15_P_4_). The at%_final_ is the at% ^18^O of soil water at the beginning of incubation (20% in this study). In addition, an assumption that O in new DNA only derived from water was made [29]. Because of the short incubation time, the mortality of newly produced ^18^O-labeled microbial cells is negligible in this study. Then microbial growth rate (ng C g^−1^ h^−1^) was calculated by multiplying the DNA production rate and *f*_DNA_ as follows:


(2) \begin{equation*} \mathrm{Growth}=\frac{f_{DNA}\ast{DNA}_{produced}\ast 1000}{DW\ast t} \end{equation*}

where *f*_DNA_ is a conversion factor indicating the ratio of soil MBC to soil DNA content (μg g^−1^ soil), DW (g) is the dry weight of soil and t is the incubation time (h). Microbial respiration rate (Respiration, ng C g^−1^ h^−1^) was calculated as the following equation:


(3) \begin{equation*} \mathrm{Respiration}=\frac{R_s}{DW\ast t}\ast \frac{p\ast n}{R\ast T}\ast V\ast 1000 \end{equation*}

where p is the atmosphere pressure (kPa), n is the molecular mass of the element C (12.01 g mol^−1^), R is the ideal gas constant (8.314 J mol^−1^ K^−1^), and T is the absolute temperature of the gas (288.15 K). V is the headspace volume (L) of the vials. R_S_ (ppm) is the CO_2_ concentration produced during the 24 h incubation period. Soil microbial CUE was calculated as the ratio of microbial growth rate over microbial total C uptake rate (Growth + Respiration) with the following equation:


(4) \begin{equation*} \mathrm{CUE}=\frac{Growth}{Growth+ Respiration} \end{equation*}

### Sequencing and data processing

The DNA samples were measured using an Illumina Miseq PE300 high throughput sequencing platform (Illumina, San Diego, CA, USA). The primer pairs 515F (5′- GTG CCAGCM GCC GCG GTA A -3′)/806R (5′- GGA CTA CHV GGG TWT CTA AT-3′) and ITS1 (5′- CTT GGT CAT TTA GAG GAA GTA A -3′)/ITS2 (5′- TGCGTT CTT CAT CGA TGC-3′) were used to amplify the V4-V5 region of bacterial 16S ribosomal ribonucleic acid genes, and the ITS1 region of fungal ITS genes, respectively. The sequencing data of soil bacteria and fungi have been deposited to the Science Data Bank (https://doi.org/10.57760/sciencedb.13692). The sequences were clustered into operational taxonomic units (OTUs) at a similarity level of 97% by UPARSE method [34]. Data were rarified to 20 981 OTUs for bacteria and 8542 OTUs for fungi across all samples, forming OTU tables as well as taxonomic OTU tables. The microbial alpha diversity (i.e. species richness, Shannon index) and β-diversity for bacteria and fungi were calculated based on OTU tables. Alpha diversity was used to measure the richness of microbial species in a single sample. Species richness and Shannon index were calculated using the diversity function in “Vegan” R package [35]. Microbial β-diversity was used to measure the similarity of microbiota composition among different samples. We used permutational multivariate analysis of variance (PERMANOVA) based on Bray–Curtis to analyze the β-diversity by “Vegan” R package for principal coordinate analysis (PCoA) sample representation.

### Network construction and analysis

Bacterial–fungal co-occurrence networks were individually constructed to infer variation in microbial community across ecological niches for AM non-rhizosphere, AM rhizosphere, ECM non-rhizosphere and ECM rhizosphere. SparCC was employed to construct the co-occurrence networks by calculating sparse correlations between microbial OTUs [36], and implemented through the “SpiecEasi” R package. To reduce the presence of rare OTUs within the dataset, bacterial OTUs occurring in <80% of the total samples and fungal OTUs occurring in <50% of the total samples were excluded. After filtration and integration, SparCC analysis was executed employing compositionality-robust correlations derived from the median of 20 iterations. Pseudo *P*-values were inferred through utilization of 100 bootstrap samples. Only robust (|r| > 0.65) and statistically significant (*P* < .05) correlations were incorporated into the network analyses. Then, we calculated topological properties for each network using the “igraph” R package [37]. Network visualization was generated with Gephi (v0.9.4) [38]. The nodes in these networks represent OTUs and the edges that connect these nodes represent correlations between OTUs. We used Gephi and conducted KS analysis to further explore distinctions among the various networks. The microbial network topological properties used in this analysis included nodes (n), links (L), average connectivity (avgK), average weighted degree, diameter, average path length, density, modularity, number of communities, clustering coefficient (CC), eigenvector centrality and centralization of degree (CD). In each network, we analyzed the main species composition of the first three modules with high modularity.

### Statistical analyses

Soil stoichiometric imbalance (R_C:N_)/(B_C:N_) was calculated based on stoichiometric characteristics of soil C:N (DOC/TDN; R_C:N_) and biomass C:N (MBC:MBN; B_C:N_) [39]. We assessed the normal distribution of data using the Shapiro–Wilk test before applying parametric methods, and log transformed if necessary. Two-way ANOVA was used to test the effects of mycorrhizal types, soil sampling position (rhizosphere and non- rhizosphere) and their interactions on soil elemental concentration, stoichiometry and microbial metabolic properties. Scheirer–Ray–Hare and Wilcoxon-test were used to analyze data with non-normal distribution of residuals. To explore which factors can better explain microbial CUE between AM and ECM, we considered microbial diversity index (richness and Shannon index), microbial network topological properties (avgK, CC, CD, and density), soil properties (pH, MBC/MBN, DOC/TDN, and microbial stoichiometric carbon/nitrogen [C/N] imbalances) as the predictors for microbial CUE. Spearman correlation was used to explore the relationship between microbial community, network topological properties, soil properties, and microbial metabolic properties using the “psych” package. Furthermore, we conducted a random forest analysis to identify the relative importance of the predictors on microbial CUE. The main goal with this random forest analysis is to reduce the number of predictors for structural equation modelling (SEM) analysis. Finally, the SEM was constructed using the “piecewiseSEM” package [40] to investigate the direct and indirect effects of main predictors on microbial CUE. Model fit statistics include the degree of freedom (df), ratio of Chi-square and df (χ^2^), probability level (*P*), R^2^ (proportion of variance explained), and AIC value.

## Results

### Soil microbial community-level physiological properties

**Figure 1:**
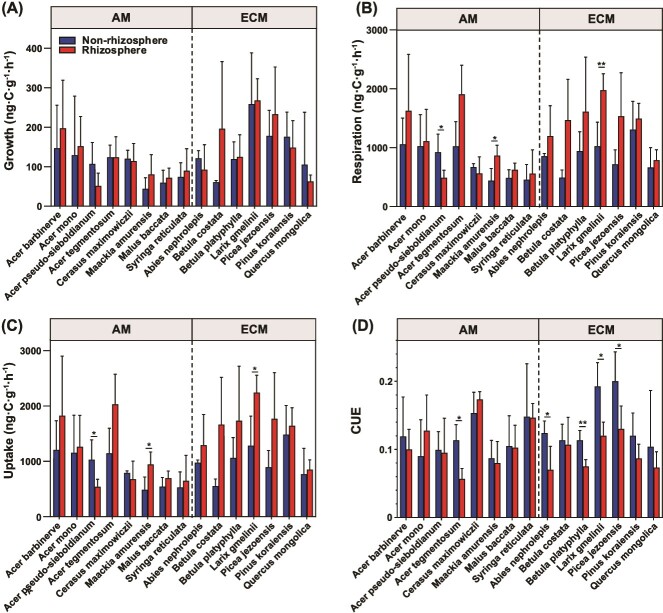
Soil microbial metabolic properties of non-rhizosphere and rhizosphere for each selected species. AM, arbuscular mycorrhizal associated trees; ECM, ectomycorrhizal associated trees. CUE, soil microbial carbon use efficiency; growth, growth rate; respiration, respiration rate; uptake, uptake rate. Error bars represent standard errors of means. Significant differences between non-rhizosphere and rhizosphere soil are indicated by ^*^0.01 < *P* ≤ .05; ^*^^*^0.001 < *P* ≤ .01; ^*^^*^^*^P ≤ .001.

The growth rate of free-living microbial communities ranged from 15.9 to 392.4  ng C g^−1^ h^−1^ across all selected tree species (Fig. 1A). However, no significant differences in microbial growth rate were observed between rhizosphere and non-rhizosphere soils at the species level (Fig. 1A). Significant differences in microbial respiration were detected for *Acer pseudo-sieboldianum*, *M. amurensis*, and *L. gmelinii* (Fig. 1B). Additionally, microbial CUE showed significant differences between rhizosphere and non-rhizosphere soils for *Acer tegmentosum* (AM), *Abies nephrolepis* (ECM), *B. platyphylla* (ECM), *Picea jezoensis* (ECM), and *L. gmelinii* (ECM) (Fig. 1D).

**Figure 2:**
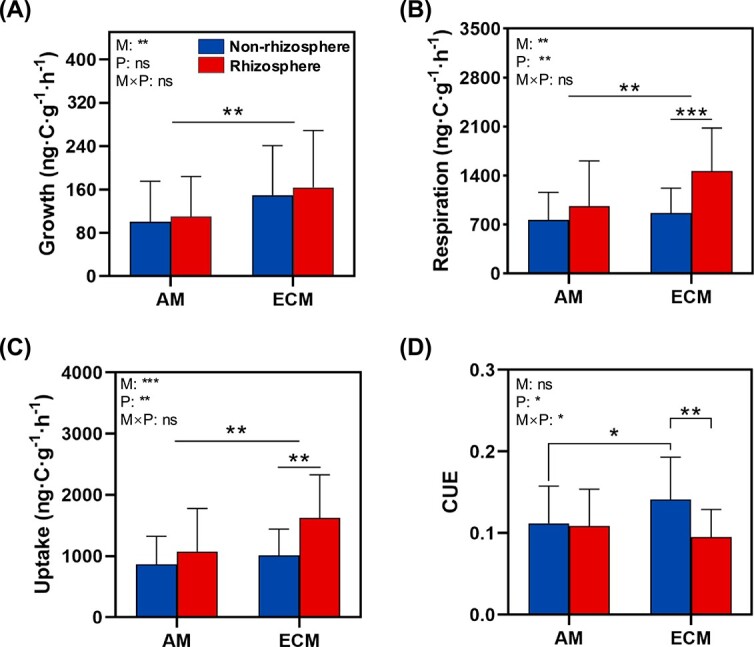
Soil microbial metabolic properties of non-rhizosphere and rhizosphere for AM and ECM trees. The four panels represent (A) microbial growth rate, (B) respiration rate, (C) carbon uptake rate, and (D) CUE. Error bars represent standard errors. Significant differences between non-rhizosphere soil and rhizosphere are indicated by ^*^0.01 < *P* ≤ .05; ^*^^*^0.001 < *P* ≤ .01; ^*^^*^^*^P ≤ .001. M, mycorrhizal type; P, soil sampling position (non-rhizosphere vs. rhizosphere). The “×” indicates interaction of M and P.

When tree species were grouped by mycorrhizal types, microbial growth, respiration, and nutrient uptake rates were generally higher under ECM compared to AM-associated trees (Fig. 2 and Table S1). Similarly, microbial CUE in non-rhizosphere soils was higher in ECM-associated trees than in AM-associated trees (Fig. 2). In contrast, microbial respiration per unit MBC (qCO₂) and nutrient uptake rate per unit MBC (qUptake) were higher in rhizosphere soils than those in non-rhizosphere soils (Fig. S1). However, microbial growth rate per unit MBC (qGrowth) and turnover rate showed no significant differences between rhizosphere and non-rhizosphere soils for both AM and ECM trees (Fig. S1).

### Soil microbial community diversity and networks

**Figure 3:**
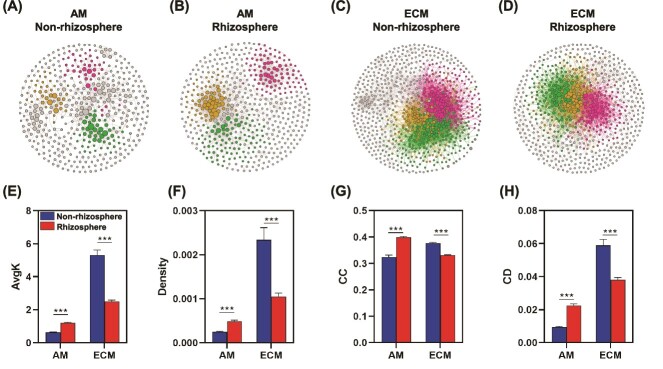
Soil microbial community networks of non-rhizosphere and rhizosphere for AM and ECM trees. Network diagram with nodes colored according to the main modules of microbial community for (A) non-rhizosphere of AM trees, (B) rhizosphere of AM trees, (C) non-rhizosphere of ECM trees, and (D) rhizosphere of ECM trees. Each node represents an individual OTU. Details of network topological attributes are listed in Supplementary Table S3. Microbial network topological properties including avgK, density, CC, and CD. Significant differences between non-rhizosphere and rhizosphere soil are indicated by ^*^0.01 < *P* ≤ .05; ^*^^*^0.001 < *P* ≤ .01; ^*^^*^^*^P ≤ .001.

Soil fungal diversity, measured by observed species richness and the Shannon index, was higher in AM-associated trees compared to ECM-associated trees (Fig. S2 and Table S2). However, this pattern was not observed for bacterial diversity. PCoA revealed no significant differences in bacterial or fungal community composition between rhizosphere and non-rhizosphere soils for both AM- and ECM-associated trees (Fig. S3). In addition, microbial networks associated with ECM trees were more complex than those of AM trees, as indicated by a higher number of nodes, edges, average degree, and network density (Fig. 3 and Table S3). Interestingly, rhizosphere microbial networks were more complex than non-rhizosphere networks in AM-associated trees, whereas the opposite trend was observed for ECM-associated trees (Fig. 3). Further, the dominant species within microbial networks differed markedly between AM- and ECM-associated trees (Fig. S4).

### Soil biogeochemical properties

**Figure 4:**
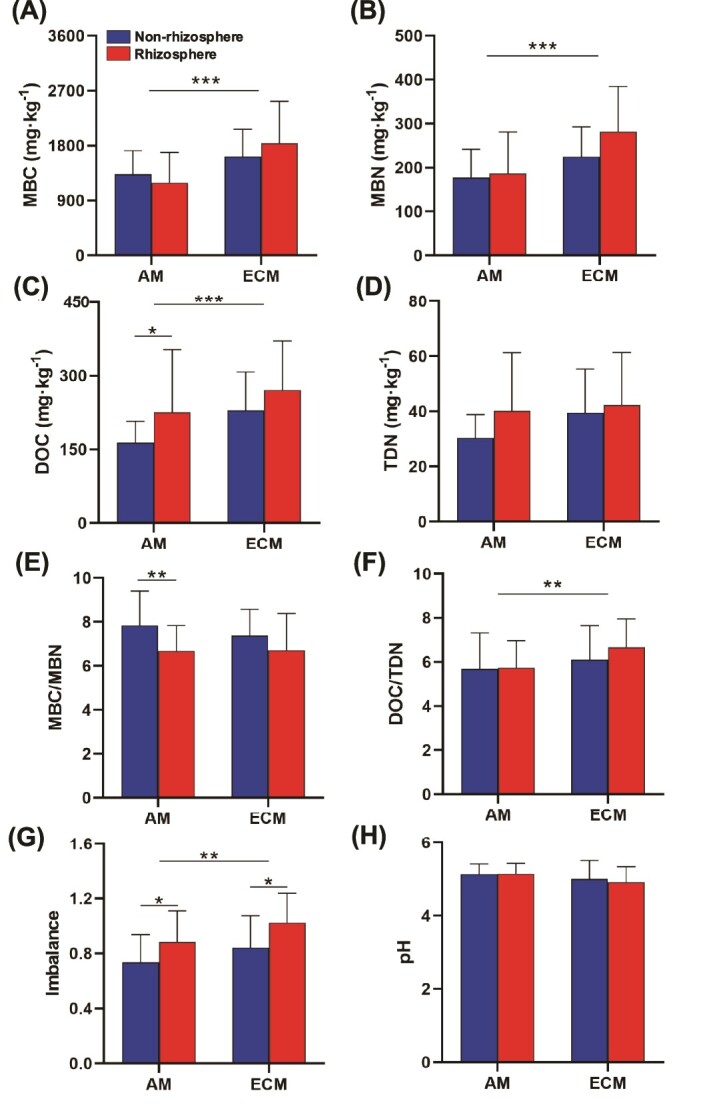
Soil and microbial properties in non-rhizosphere and rhizosphere soils of AM and ECM. DOC, dissolved organic carbon; TDN, total dissolved nitrogen; Imbalance = (DOC/TDN) / (MBC/MBN). Error bars represent standard errors of means. Significant differences (Wilcoxon test) between non-rhizosphere and rhizosphere soils are indicated by ^*^0.01 < *P* ≤ .05; ^*^^*^0.001 < *P* ≤ .01; ^*^^*^^*^P ≤ .001.

Soil MBC and MBN were higher in ECM than those in AM-associated trees, and consequently, there was no significant difference in the MBC/MBN ratio between ECM and AM trees (Fig. 4 and Table S2). Since the DOC/TDN ratio was higher in ECM than AM, the microbial stoichiometric C/N imbalance significantly differed between AM-associated and ECM-associated trees (Fig. 4). Soil pH values showed no difference between AM-associated and ECM-associated trees (Fig. 4).

### Influencing factors on microbial physiological properties

**Figure 5:**
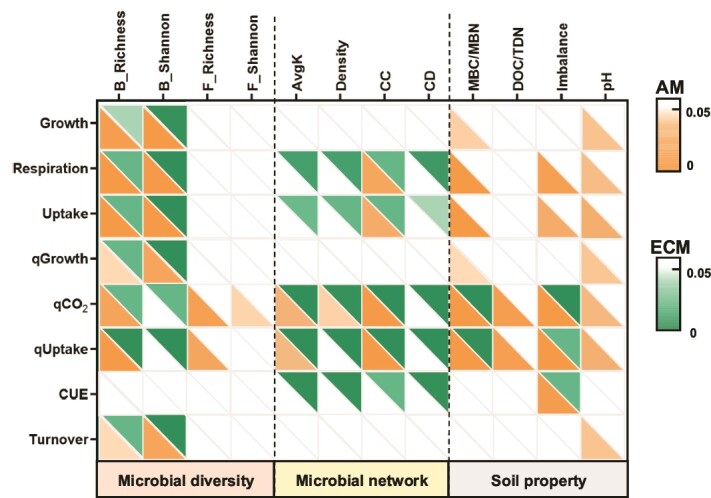
Correlation between microbial metabolism processes and influencing factors. Spearman correlations of these variables are shown with a color gradient denoting significant (*P* ≤ .05). Soil microbial metabolism processes include microbial growth rate (growth), respiration rate (respiration), uptake rate (uptake), microbial growth rate per unit MBC (qGrowth), microbial respiration rate per unit MBC (qCO_2_), microbial C uptake rate per unit MBC (qUptake), microbial carbon use efficiency (CUE) and turnover rate (turnover). Microbial diversity indices include bacterial OTU richness (B_richness), bacterial Shannon index (B_Shannon), fungal OTU richness (F_richness), and fungal Shannon index (F_Shannon). Microbial network topological indices include avgK, density, CC, and CD. Microbial stoichiometric imbalance (imbalance) was calculated as the ratio of soil C/N ratio over microbial biomass C/N ratio. MBC/MBN, the ratio of microbial biomass carbon over nitrogen; DOC/TDN, soil dissolved carbon over nitrogen; pH, soil pH.

For ECM-associated trees, correlation analysis showed that microbial network topological indices (e.g. avgK, network density, CC, and CD) were positively correlated with microbial CUE (Fig. 5 and Fig. S5). Additionally, microbial CUE was negatively correlated with soil microbial stoichiometric C/N imbalances in ECM-associated soils (Fig. 5). In contrast, for AM-associated soils, microbial CUE was negatively correlated with stoichiometric imbalances of C/N, but network topological indices were not significantly associated with microbial CUE (Fig. 5). Fungal diversity, MBC/MBN, DOC/TDN, and pH were not correlated with any microbial C physiological parameters in ECM-associated soils, whereas in AM-associated soils, pH was correlated with all microbial physiological parameters except CUE (Fig. 5 and Fig. S5).

**Figure 6:**
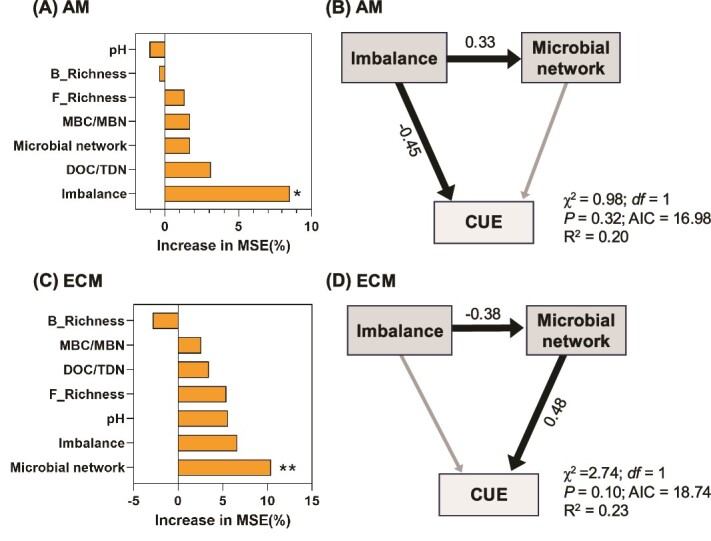
Random forest model and structural equation modeling for describing the effects of predictors on microbial CUE. Microbial network topological index and microbial stoichiometric imbalance were selected as two predictors of microbial CUE from random forest. The bar plots showing the relative effects of soil properties, microbial diversity, microbial network topological index (avgK), and microbial stoichiometric imbalance (imbalance) on microbial CUE by using random forest models for (A) AM and (C) ECM trees. The SEM showing how predictors’ direct and indirect influencing on microbial CUE for (B) AM and (D) ECM trees. Grey arrows in SEM indicate non-significant effects of the predictors on CUE. The number beside the arrow is the corresponding standardized coefficient.

Random forest analysis identified microbial stoichiometric C/N imbalance as the strongest predictor of microbial CUE in AM-associated soils, whereas microbial network complexity emerged as the strongest predictor in ECM-associated soils (Fig. 6). Structural equation modeling further revealed distinct mechanisms driving microbial CUE: in AM-associated soils, microbial CUE was directly influenced by microbial stoichiometric C/N imbalances, while in ECM-associated soils, microbial stoichiometric imbalances indirectly influenced microbial CUE through their effects on network complexity (Figs 6 and S6).

## Discussion

Contrary to our initial hypothesis, our study revealed that the soil microbial CUE of ECM trees in the non-rhizosphere is higher than that of AM trees. This can be attributed to a higher microbial growth rate associated with ECM soils compared to AM soils, while the microbial respiration showed negligible differences in the non-rhizosphere soil between ECM and AM (Figs 1 and 2). This observation is in line with previous studies that reported differences in microbial CUE between soils associated with AM and ECM trees [41, 42]. Furthermore, our study showed that the MBC is higher in soils associated with ECM trees compared to those associated with AM trees (Fig. 4). These findings collectively provide new evidence supporting the higher soil carbon storage in ECM trees compared to AM trees, potentially due to an enhanced microbial carbon use and turnover pathway [14, 43]. This indicates that the exudates released into the soil by ectomycorrhizal hyphae could be more readily utilized by microorganisms, thereby contributing more microbially-derived carbon to soils [6]. There is growing evidence that microbial residues and necromass play an important role in forming stable soil organic matter [14, 24], contributing more to mineral-associated carbon compared to plant inputs [44, 45]. Our findings highlight that the mycorrhizal plants in the studied forest may control soil carbon storage by regulating the metabolic processes of free-living microorganisms as well as their necromass production.

In terms of microbial diversity, our study revealed that bacterial diversity was unaffected by mycorrhizal types or sampling position (rhizosphere vs. non-rhizosphere). However, fungal diversity was higher in AM-associated soils compared to ECM-associated soils (Figs S2 and S3). This pattern may result from faster litter decomposition rates in AM ecosystems and the complex interactions between ECM fungi and free-living microorganisms [12, 27]. Network analysis further indicated that microbial networks in ECM soils were more complex, characterized by higher connectivity and CD (Fig. 3). These differences in network properties could be driven by variations in plant litter types and soil nitrogen availability [12].

A notable finding of our study is the differential factors influencing microbial CUE in AM and ECM systems (Fig. 6). In AM soils, the stoichiometric C/N imbalance emerged as the primary predictor of microbial CUE. AM fungi enhance nutrient acquisition, particularly nitrogen, which can alleviate nutrient limitations for microbes and reduce the metabolic costs associated with nutrient uptake [22, 27]. This nutrient availability may lead to higher microbial CUE by lowering respiration costs and increasing growth efficiency. Conversely, ECM soils exhibited higher microbial stoichiometric imbalance of C/N, indicative of nitrogen limitation (Figs 4 and S6). This imbalance may result from competition between ECM fungi and free-living microbes for organic nitrogen, constraining microbial anabolism and lowering CUE [8, 27].

In ECM soils, microbial network complexity was the dominant factor influencing CUE (Fig. 6). Our analysis revealed that ECM soils had more intricate microbial networks, characterized by higher connectivity and CD (Fig. 3). Such networks may enhance metabolic efficiency through synergistic interactions, where certain microbial taxa specialize in decomposing complex organic materials into simpler compounds that others can readily utilize [15, 31, 46]. ECM fungi, with their extensive hyphal networks, further contribute to this complexity and F:B ratio by accessing and decomposing recalcitrant organic matter [11, 44]. These findings suggest that microbial network properties, rather than stoichiometric constraints, play a more critical role in determining microbial CUE in ECM systems.

Our study also challenges the conventional assumption that microbial diversity is the primary driver of microbial CUE [47]. While fungal diversity was higher in AM-associated soils, no significant correlation was observed between microbial diversity and CUE. Instead, the complexity and structure of microbial networks, particularly in ECM soils, were positively correlated with CUE (Fig. 5). This aligns with emerging evidence suggesting that microbial interactions, rather than species diversity alone, are crucial for ecosystem processes [15]. By advancing the understanding of microbial networks, our study underscores the importance of considering microbial interactions in predicting soil carbon turnover and storage.

Despite providing valuable insights, our study has limitations. The disruption of fungal hyphae during soil sampling may have affected microbial community structure and activity, potentially influencing measurements of microbial growth, respiration, and CUE. Future research should focus on developing in situ methods for assessing microbial CUE to preserve the natural structure and function of microbial communities. Additionally, as our study was conducted in a temperate forest, further investigation is needed to explore whether the observed patterns hold across different ecosystems.

In summary, our study demonstrates significant differences in microbial CUE between AM- and ECM-associated trees, with ECM non-rhizosphere soils exhibiting higher CUE. These differences are primarily driven by stoichiometric C/N imbalance in AM systems and microbial network complexity in ECM systems. Our findings provide a mechanistic basis for understanding variations in soil carbon storage between mycorrhizal types and highlight the need to integrate microbial network properties into soil carbon models. Exploring how shifts from ECM- to AM-dominated vegetation under climate change scenarios may alter soil microbial processes and carbon storage is crucial for predicting ecosystem carbon dynamics. Incorporating microbial parameters, such as network complexity and stoichiometric imbalance, into predictive models could significantly improve our understanding of soil carbon sequestration in temperate forest.

## Supplementary Material

2_Supplementary_final_ycae173

## Data Availability

All data are available in the main text or the supplementary materials. The raw sequences of bacteria and fungi can be accessed as the link: https://doi.org/10.57760/sciencedb.13692.
