# Supplementary material for: Soil microbial carbon use efficiency differs between mycorrhizal trees: insights from substrate stoichiometry and microbial networks
Source: ISME Commun. 2024 Dec 27;5(1):ycae173. doi: 10.1093/ismeco/ycae173 (PMC11742255; doi:10.1093/ismeco/ycae173)
Supplement: 2_Supplementary_final_ycae173 [file 2_supplementary_final_ycae173.pdf]

Supplementary information for

Soil microbial carbon use efficiency differs between mycorrhizal trees: insights from substrate stoichiometry and microbial networks

Jing Yu [1, 2, 4], Jingyi Yang [1,4], Lingrui Qu [1], Xiaoyi Huang[1,2], Yue Liu [1], Ping Jiang [1], Chao Wang [1, 3,*]

[1] CAS Key Laboratory of Forest Ecology and Silviculture, Institute of Applied Ecology, Chinese Academy of Sciences, Shenyang, 110016, China

[2] University of Chinese Academy of Sciences, Beijing, 100049, China

[3] Key Laboratory of Terrestrial Ecosystem Carbon Neutrality, Liaoning Province, Shenyang, 110016, China

[4] These authors contribute equally to this work

***Corresponding author**

Chao Wang

Institute of Applied Ecology, Chinese Academy of Sciences

No. 72 Wenhua Road, Shenyang, Liaoning

110016, China

Telephone: +86-24-83970570

Fax: +86-24-83970300

Email: cwang@iae.ac.cn

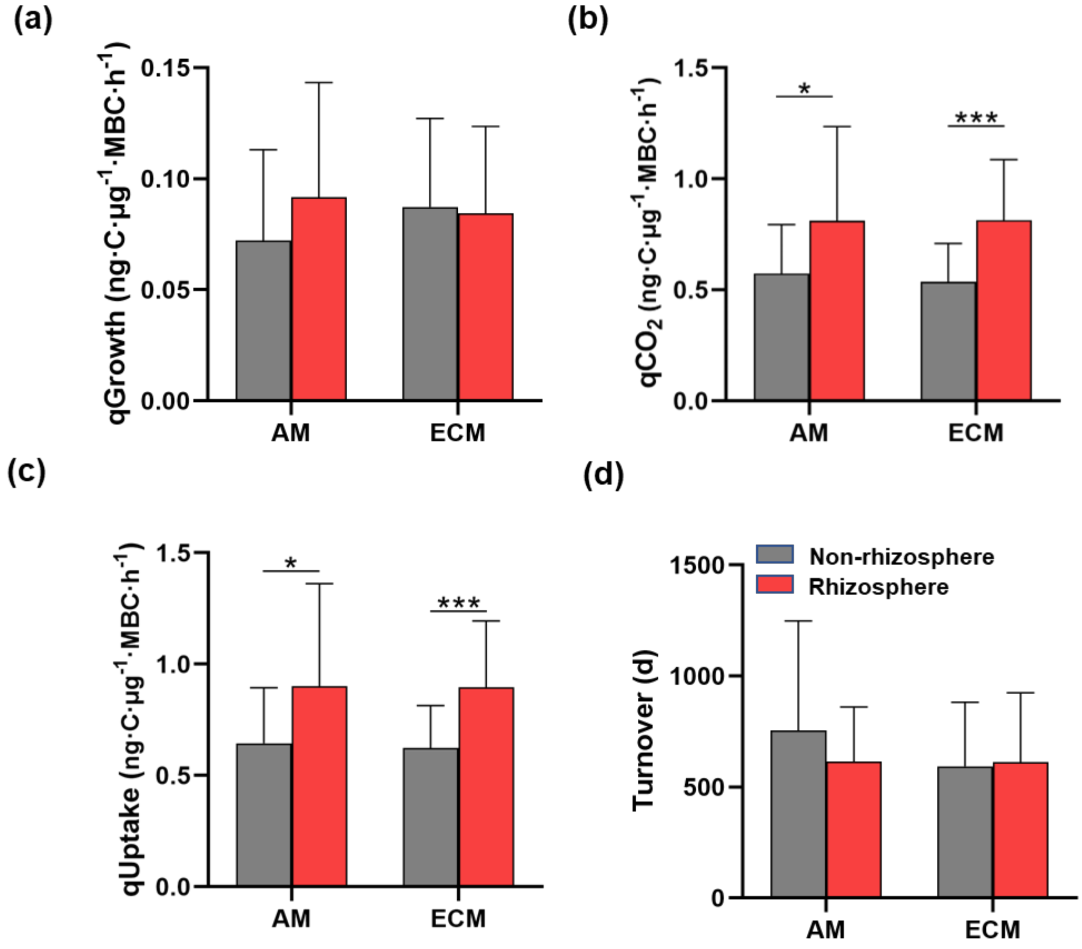

Figure S1. Soil microbial function indicators per unit MBC in non-rhizosphere and rhizosphere soils of AM and ECM. AM, arbuscular mycorrhizal associated trees; ECM, ectomycorrhizal associated trees. The four panels represent microbial growth rate per unit MBC (a), metabolic quotient (b), microbial C uptake rate per unit MBC (c) and microbial turnover rate (d). Error bars represent standard errors of means. Significant differences between non-rhizosphere and rhizosphere soil are indicated by *, $0.01 < P \leq 0.05$; **, $0.001 < P \leq 0.01$; ***, $P \leq 0.001$.

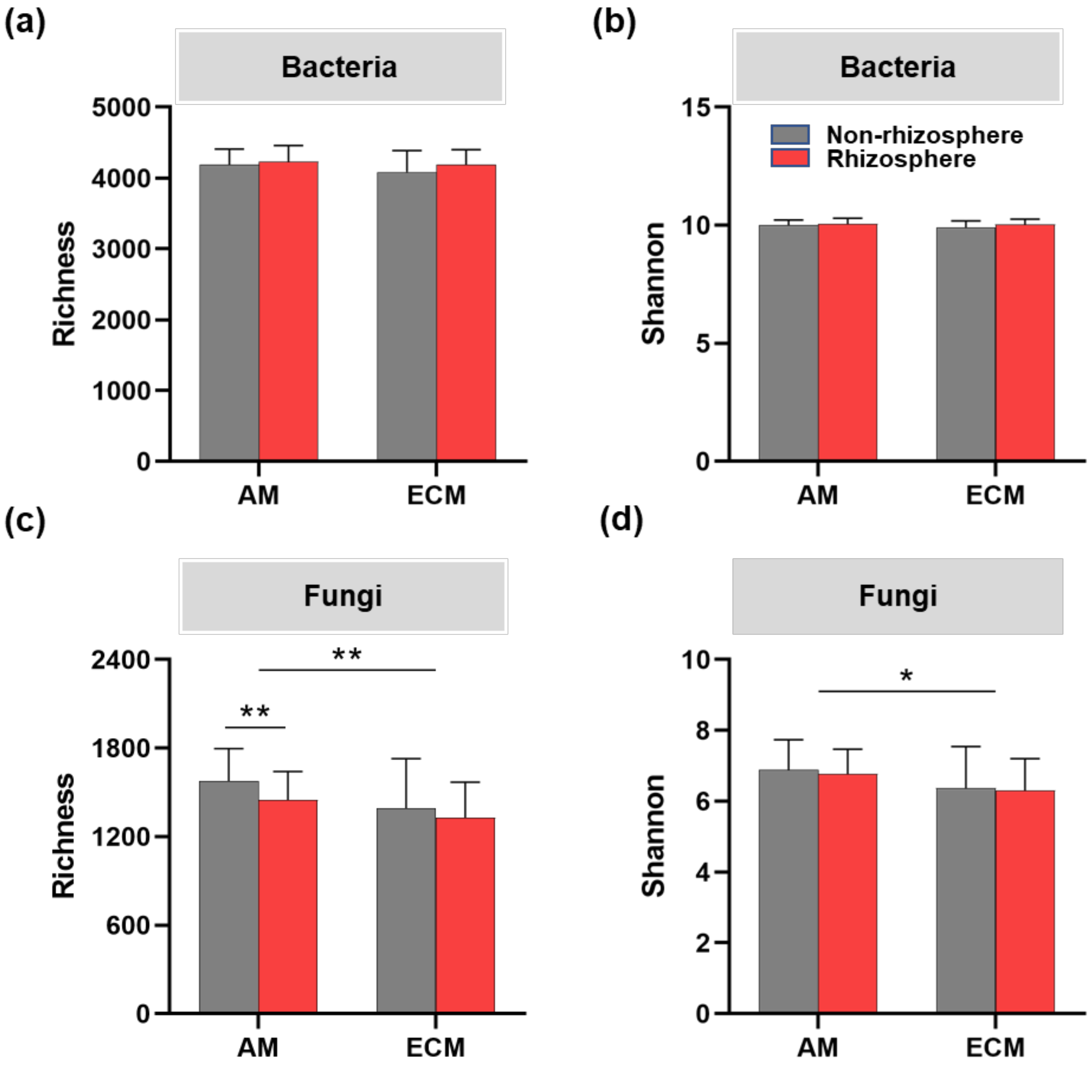

Figure S2. Microbial alpha diversity indices in non-rhizosphereand rhizosphere soils of AM and ECM. AM, arbuscular mycorrhizal associated trees; ECM, ectomycorrhizal associated trees. Richness, observed species richness; Shannnon, Shannon's diversity index. Error bars represent standard errors of means. Significant differences between non-rhizosphere and rhizosphere soil are indicated by *, $0.01 < P \leq 0.05$; **, $0.001 < P \leq 0.01$; ***, $P \leq 0.001$.

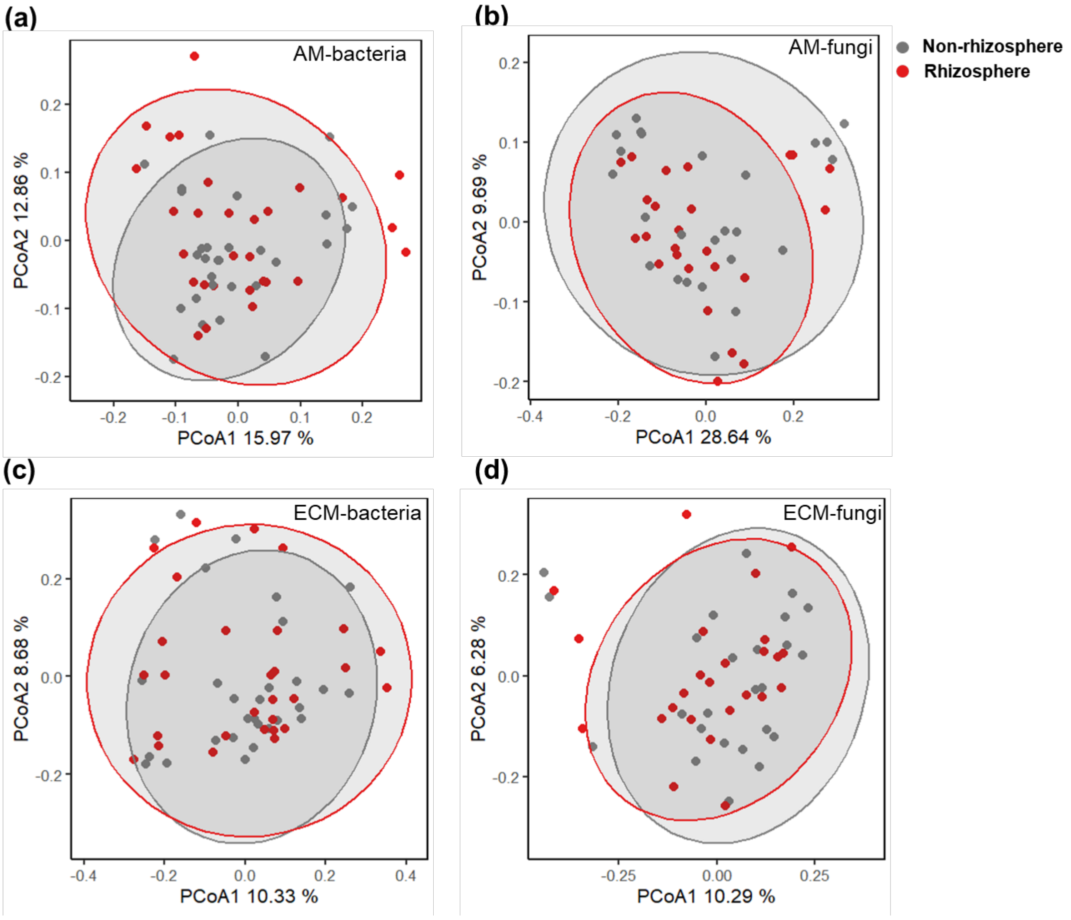

Figure S3. Soil microbial β diversity. AM, arbuscular mycorrhizal associated trees; ECM, ectomycorrhizal associated trees. Unconstrained principal coordinates analysis (PCoA, for principal coordinates PCoA1 and PCoA2) with Bray-Curtis distance showing that bacterial and fungal composition of soil are significantly different in different filed ($P < 0.001$, permutational multivariate analysis of variance (PERMANOVA) by Adonis). (a-b) Dissimilarity of bacterial communities between non-rhizosphere and rhizophere soils in AM (a) and ECM (b). (c-d) Dissimilarity of fungal communities between non-rhizosphere and rhizophere soil in d AM (c) and ECM (d). PCoA, principal coordinate analysis.

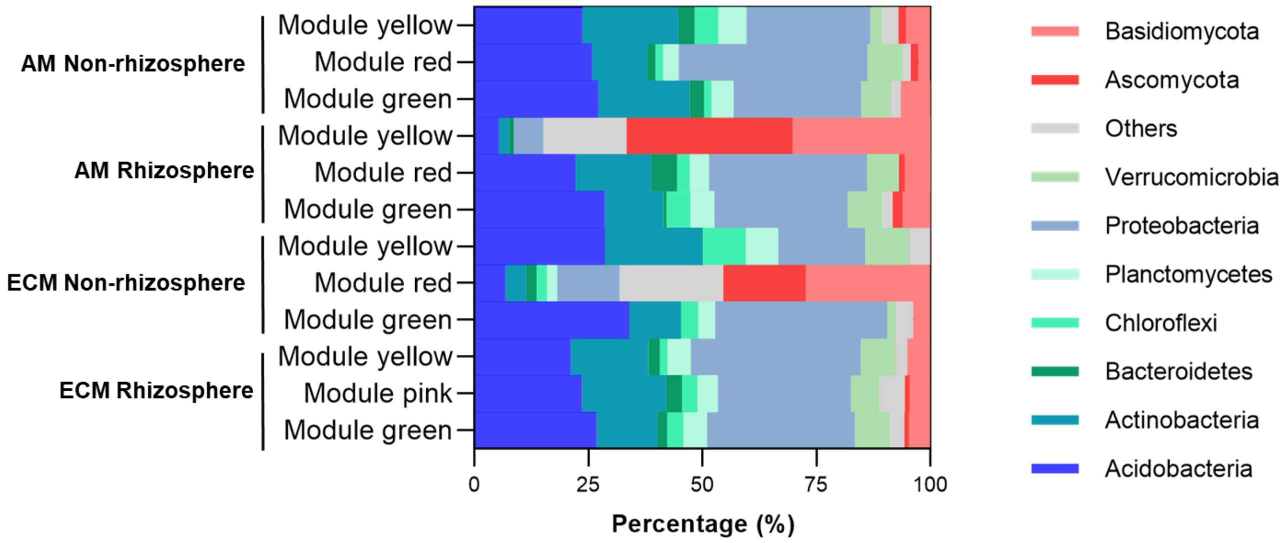

Figure S4 Microbial taxa number and their relative proportions are analyzed in each module for the microbial network. AM Non-rhizosphere, non-rhizosphere soils for arbuscular mycorrhizal associated trees; AM Rhizosphere, rhizosphere soils for arbuscular mycorrhizal associated trees; ECM Non-rhizosphere, non-rhizosphere soils for arbuscular mycorrhizal associated trees; ECM Rhizosphere, rhizosphere soils for arbuscular mycorrhizal associated trees; Fungal phyla include *Ascomycota* and *Basidiomycota*. Bacterial phyla include *Acidobacteria*, *Actinobacteria*, *Bacteroidetes*, *Chloroflexi*, *Planctomycetes*, *Proteobacteria*, *Verrucomicrobia* and others.

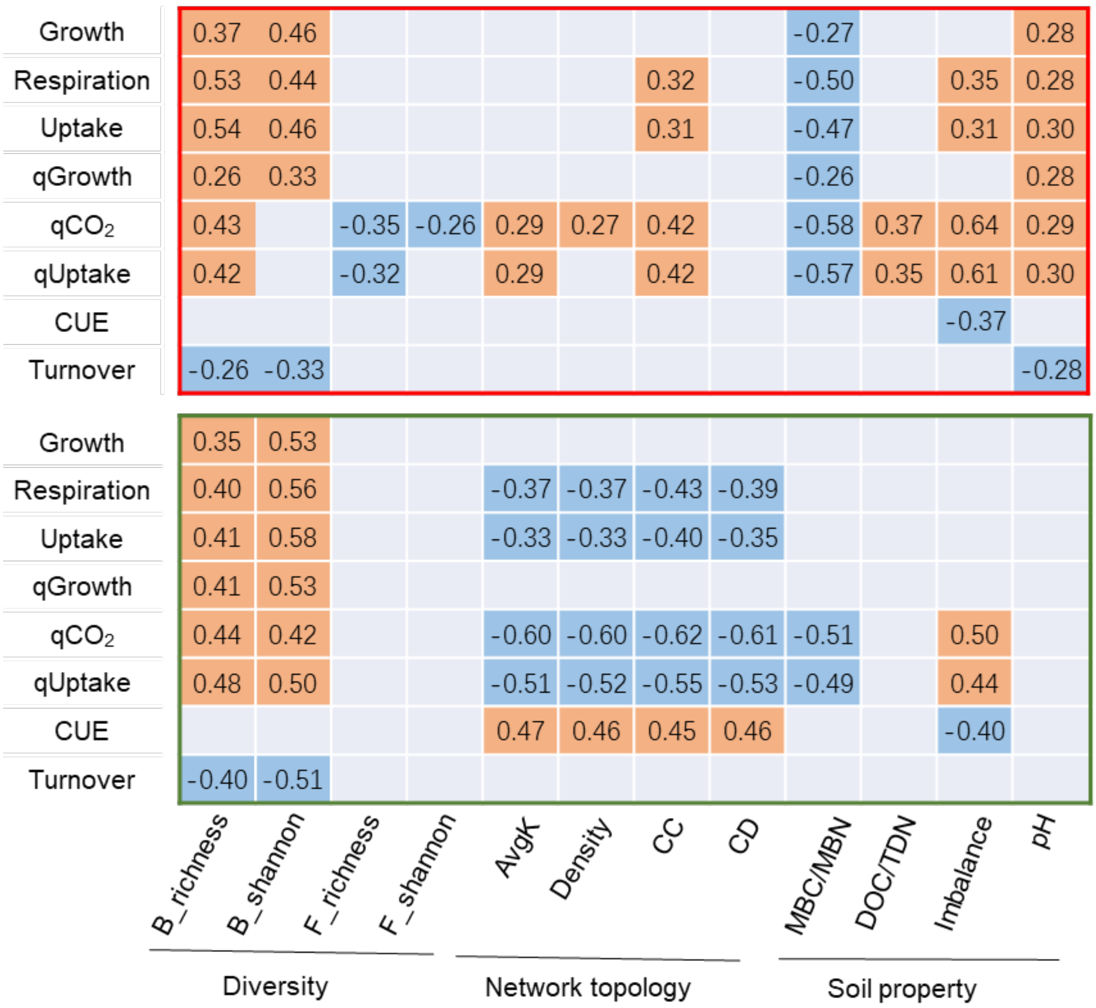

Figure S5. Spearman correlation between soil microbial C metabolism processes and microbial as well as soil properties. AM, arbuscular mycorrhizal associated trees (red color); ECM, ectomycorrhizal associated trees (green color). Significant ($P \leq 0.05$) correlations are shown here, with orange for positive correlations and blue for negative correlations. Numbers inside cells are corresponding correlation coefficients. Correlations with $P > 0.05$ are marked in grey.

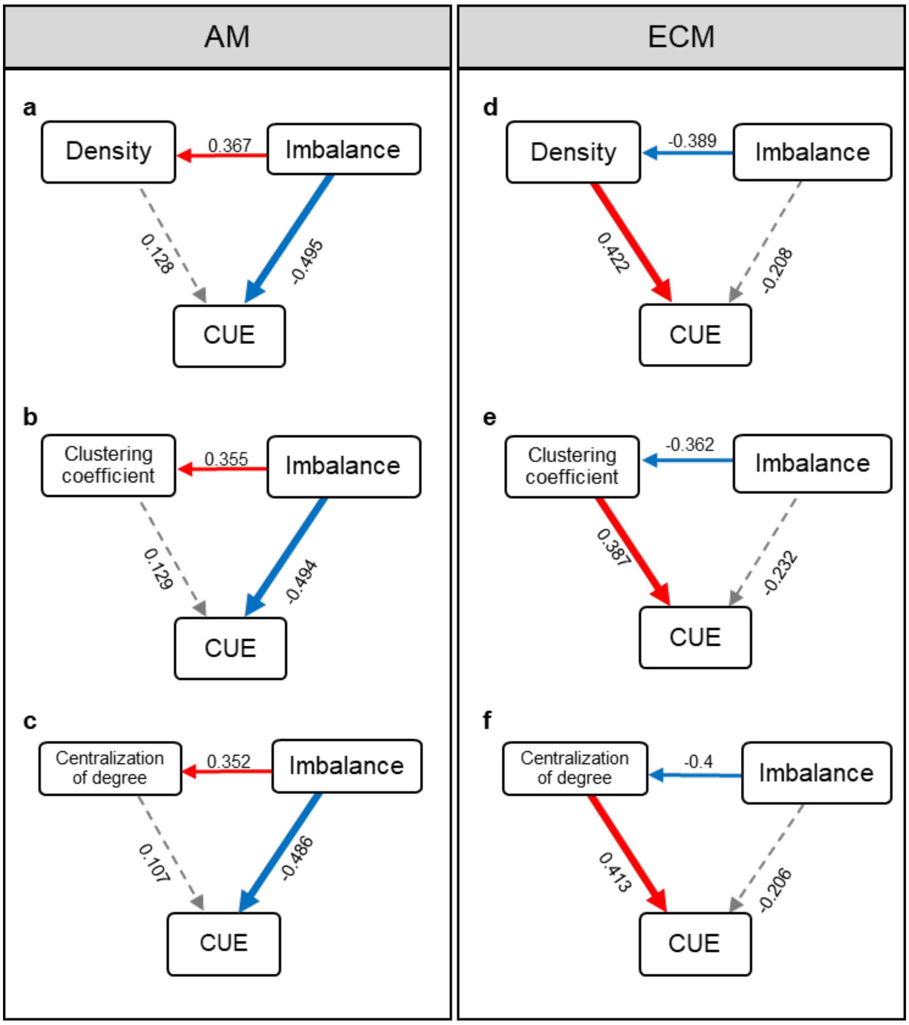

Figure S6. Structural equation model describing the effects of predictors on CUE for AM and ECM. AM, arbuscular mycorrhizal associated trees; ECM, ectomycorrhizal associated trees. Structural equation model (SEM) of topology (microbial network topology indices) and imbalance as predictors of CUE (soil microbial carbon use efficiency). The microbial network topology indices including density, clustering coefficient and centralization of degree. Single-headed arrows indicate a one-way directed relationship. Solid arrows indicate statistically signifcant ($P < 0.05$) positive (red) or negative (blue) effects of the predictors on CUE, while dashed gray arrows indicate statistically non-signifcant. The number beside the arrow is the corresponding standardized coefficient (r).The width of arrows is proportional to the strength of standardized path coeffcients.

Table S1. Two-way ANOVA analysis of microbial carbon metabolism parameters. Statistical significances of mycorrhizal type (i.e, AM and ECM), soil position (i.e, non-rhizosphere and rhziosphere) and their interaction as explanatory variables of microbial growth rate, respiration rate, uptake rate, soil microbial carbon use efficiency (CUE), growth rate per unit MBC (qGrowth), metabolic quotient (qCO_2), C uptake rate per unit MBC (qUptake) and turnover rate (turnover). All resource terms were log transformed to meet assumptions of normality. Significant ($P < 0.05$) results are highlighted in bold. "×" indicate interaction of two predictors.

	**Mycorrhizal type (M)**	**Sample position (P)**	**M×P**
Growth	0.003	0.434	0.828
RS	0.001	0.001	0.084
Uptake	<0.001	0.003	0.140
CUE	0.351	0.016	0.028
qGrowth	0.331	0.206	0.144
qCO_2	0.875	<0.001	0.468
qUptake	0.763	<0.001	0.685
Turnover	0.341	0.199	0.145

Table S2. Scheirer-Ray-Hare analysis of soil properties and microbial diversity. Significant (*P* < 0.05) results are highlighted in bold. "×" indicate interaction of two predictors.

	**Mycorrhizal type (M)**	**Soil position (P)**	**M×P**
MBC	<0.001	0.694	0.161
MBN	<0.001	0.386	0.186
MBC/MBN	0.520	<0.001	0.501
DOC	<0.001	0.004	0.526
TDN	<0.001	0.140	0.364
DOC/TDN	0.005	0.311	0.543
C/N Imbalance	0.005	<0.001	0.976
Richness_B	0.118	0.270	0.269
Shonnon_B	0.260	0.070	0.250
Richness_F	0.005	0.004	0.658
Shonnon_F	0.017	0.243	0.906

Table S3. Microbial network topological properties.

Properties	AM non-rhizosphere	AM rhizosphere	ECM non-rhizosphere	ECM rhizosphere
n	487	544	807	690
L	873	1559	6242	3088
Average degree	3.585	5.732	15.47	8.951
Average weighted degree	2.508	4.019	10.844	6.235
Diameter	15	13	16	18
Average path length	5.66	5.098	4.825	4.693
Density	0.007	0.011	0.019	0.013
Modularity	0.797	0.654	0.413	0.488
Number of communities	54	63	29	42
Average clustering coefficient	0.429	0.509	0.54	0.465
Eigenvector centrality	0.043	0.024	0.009	0.012
Green	#1	#4	#3	#0
Red	#26	#11	#1	#1
Yellow	#9	#6	#2	#4
